# The role of bone health in low-velocity fractures and the effects of obesity on the growing skeleton

**DOI:** 10.1016/j.jposna.2024.100053

**Published:** 2024-04-09

**Authors:** Adam Kreutzer, Jessica McMichael, Philip Nowicki

**Affiliations:** 1 Department of Orthopaedic Surgery, Riverside University Health System Medical Center, Moreno Valley, CA, USA; 2 Children’s Hospital of Orange County, Orange, CA, USA; 3 Helen DeVos Children’s Hospital, Grand Rapids, MI, USA; 4 Department of Surgery, Michigan State University College of Human Medicine, Grand Rapids, MI, USA

**Keywords:** Vitamin-D insufficiency, Obesity, Adipocytes, Leptin, RANKL

## Abstract

Vitamin D insufficiency in pediatric patients is common. Low levels of vitamin D affect bone mineralization, and in growing children, affect bone formation. This effect on overall bone health places children with vitamin D insufficiency and deficiency at risk for low-velocity fractures. Counseling pediatric patients on adequate vitamin D and calcium supplementation can decrease long-term bone health deficits and lead to improved health status as adults. In addition, obesity rates continue to increase in children. Obesity has direct effects on bone metabolism that negatively impact growing skeletons. Refined definitions for obesity will help prevent labeling patients inappropriately as obese but also allow physicians to counsel children and their parents on the long-term risks that obesity plays on both bone health and overall health.

**Key Concepts:**

(1) Vitamin D has a strong influence on bone health with low levels of vitamin D being associated with low bone mineralization on DEXA scans.
(2) Children with low vitamin D are at risk for low-velocity fractures including the need for surgical intervention for these lower energy injuries.
(3) Obesity leads to a chronic inflammatory state that creates a negative overall effect on the growing skeleton.
(4) Obesity can be a stigmatizing condition resulting from many factors related to socioecological, environmental, and genetic influences but when appropriately addressed, can lead to improved long-term bone and overall health.

## Introduction

Vitamin D insufficiency is common in the United States population, including the pediatric population. Given the high percentage of children with hypovitaminosis D, this article aims to review the current literature on vitamin D’s relationship to bone health, low-energy fracture risk. Research into the effects of obesity on growing bones continues to grow. A large portion of the pediatric population who are obese will enter adulthood with the health risks and concerns associated with poor long-term bone health. The second half of this article will review the most current basic science knowledge related to the effects of obesity on the growing skeleton.

## The role of bone health on low-velocity fractures

Bone health is a complex balance of bone creation and breakdown that is influenced by nutritional status, exercise, and environmental factors. When there are deficiencies in these factors, the bone weakens which leads to an increased risk of fracture from lower velocity injuries. In pediatric patients, fractures from lower velocity injuries are common which may act as an indicator of deficiencies in their bone health. Given that peak bone mineral density is achieved by the second decade of life, pediatric low bone mineral density and low bone health is a problem that may extend into adult years [1].

Vitamin D’s importance to bone health was established when found to correlate with rickets in young children and later found to cause osteomalacia in adults [2]. Since then, scientific interest in Vitamin D and its role in human health has gained significant traction. This research has found that vitamin D has multiple mechanisms by which it affects bone health. It creates osteocalcin to help bone absorb calcium, stimulates osteoblasts to activate osteoclasts encouraging bone turnover, and acts on the gut to increase calcium and phosphorus absorption [3], [4]. While the exact definitions of vitamin D insufficiency and deficiency are still debated, recent research has shown that an alarmingly high percentage of children have low vitamin D levels, ranging from 30% to 70% [2], [5], [6].

In a study on British children, fractures were common, affecting 2 out of every 100 children per year [7]. The average age of the child with a fracture was 10 years old, 61.4% of which were male [7]. The majority (82.2%) of these fractures affected the upper limb and were most often associated with a fall from below bed height [7]. The prevalence of fractures increased as age increased (2.1% from 0 to 1 year of age, 11.6% for 2 to 4 years, peaking at 51.3% for 5 to 11 years) but decreased in older children/adolescents (33.8% for 12 to 16 age group) [7]. This increased fracture risk occurs at the onset of puberty, a time where the rapid growth of the skeleton leads to decreased skeletal strength, a lag in cortical thickness, and decreased mineralization [8]. Rates of vitamin D deficiency are higher in adolescents than in younger children, also thought to be a result of the rapid increase in skeletal growth [1]. Fractures sustained at a younger age are associated with increased fracture risk later in life and maybe a sign for greater overall lifelong musculoskeletal risk [8], [9], [10]. These statistics serve to highlight the large number of fractures that are occurring in children and the low-energy mechanisms associated with them. Optimizing pediatric bone health could result in fewer lower-energy fractures and reduce the short and long-term morbidity associated with them.

Low levels of vitamin D affects bone mineralization as seen on DEXA scans [11], [12]. Multiple studies have found significantly lower levels of vitamin D in children with fractures compared to those without [13], [14], [15], [16], [17]. Ergun and Consever [18] found pediatric patients with fractures were significantly higher over age-matched controls to be vitamin D deficient. Their numbers showed a 14.8-fold higher vitamin D deficiency rate in upper extremity fractures and 2.9 times greater rate in lower extremity fractures. Associations have been found between fracture severity and hypovitaminosis D as well as fracture rates and vitamin D-specific supplementation [2], [19], [20], [21], [22]. Minkowitz et al. [23] demonstrated that vitamin D deficiency did not lead to increased fracture risk but did correlate significantly with the risk of having an operative fracture. This association between hypovitaminosis D and fracture severity suggests that patients with lower levels of vitamin D are disproportionately exposed to the risks associated with surgery.

Despite the established importance for adequate vitamin D levels and skeletal health, consensus has not been reached on what constitutes hypovitaminosis D. Multiple medical bodies have created guidelines for the diagnosis of vitamin D insufficiency and deficiency, but the proposed levels have high variability (Table 1). As vitamin D plays a central role in bone health and fracture risk, establishing specific parameters for vitamin D sufficiency in children is necessary.

**Table 1:** Table showing the normal serum vitamin D levels vs insufficiency and deficiency [24], [25].

	Normal	Insufficient	Deficient
Institute of Medicine (2010), Global Consensus (2016)	>20 ng/mL	12-19 ng/mL	<12 ng/mL

The Endocrine Society (2011), Italian Pediatric Society (2018), Polish Society of Pediatric Endocrinology and Diabetes (2018)	>30 ng/mL	20-29 ng/ mL	<20 ng/mL

Given the risks of low vitamin D, understanding what the risks factors are for deficiency can help physicians know which patients to screen. There are many factors that put individuals at higher risk for vitamin D deficiency including nutritional deficiency, skin hyperpigmentation, reduced sun exposure, seasonal effects, older age, and obesity [2]. Adolescents have a higher rate of vitamin D deficiency than younger children [1]. Further risk factors for hypovitaminosis D include medical conditions leading to decreased absorption and medications including glucocorticoids, anticonvulsants, antifungals, and antiretrovirals [1]. Routine vitamin D testing of healthy children is not currently recommended. Screening children with risks for vitamin D deficiency is left to primary care physicians and pediatric Orthopaedic surgeons who routinely see patients with low-velocity fractures.

With low-velocity fractures being common and a sign for lifelong skeletal health deficiencies, identifying and optimizing interventions early on can prevent fractures as well as long-term consequences [9]. Vitamin D has been established as an important nutrient in skeletal health, but the current literature is unclear and occasionally contradictory on what specific role it plays [2]. What is clear is that hypovitaminosis D is associated with decreased mineralization of the skeletal as seen on dual x-ray absorptiometry (Dexa) scans and that an alarmingly high percentage of children have inadequate levels of vitamin D [11], [12]. Vitamin D supplementation can improve health outcomes in these patients but further study into how much to supplement and the optimal level to supplement to is still needed [2]. Along with vitamin D, other nutrients play important roles in skeletal health, some of which are insufficient in the American diet and others of which are consumed excessively [5]. Patients with obesity suffer from low-velocity fractures as will be discussed next. Pediatricians and pediatric Orthopaedic surgeons can inquire about dietary intake and encourage as well as facilitate nutritional behavioral change [1]. The benefits of interventions in childhood can improve bone health into adulthood [25].

## The effects of obesity on the growing skeleton

Currently, pediatric obesity rates mirror the rates of adults in the developed and developing world, though childhood obesity has risen much greater than adults in recent years [26], with obesity rates tripling since the 1970s [27], [28]. Thirty-two percent of children can be classified as overweight and obese, with 18% categorized specifically as obese [29], [30]. These percentages are higher in the African American and Hispanic populations [31]. Obesity is a growing health concern affecting all ages and the consequences of conditions associated with obesity (ie, cardiovascular disease, hypertension, stroke, type 2 diabetes) will continue to produce a heavy future financial burden on health systems worldwide [32], [33], [34].

What exactly constitutes obesity remains controversial. Classification for pediatric patients is based upon the body mass index (BMI) percentile for age. The Centers for Disease Control separates BMI groups as follows: underweight (<5th percentile), normal/healthy weight (5th to 85th percentile), overweight (85th to 95th percentile), and obese (≥95th percentile) [35]. This measurement can be misleading as higher-weight individuals with greater muscle mass can be labeled as obese but physically, they are not. Failure to account for body fat composition is an additional weakness when utilizing BMI alone for health grouping [33]. “Labeling” patients as obese has potential detrimental social and mental effects, thus an accurate definition of obesity is required.

**Figure 1:**
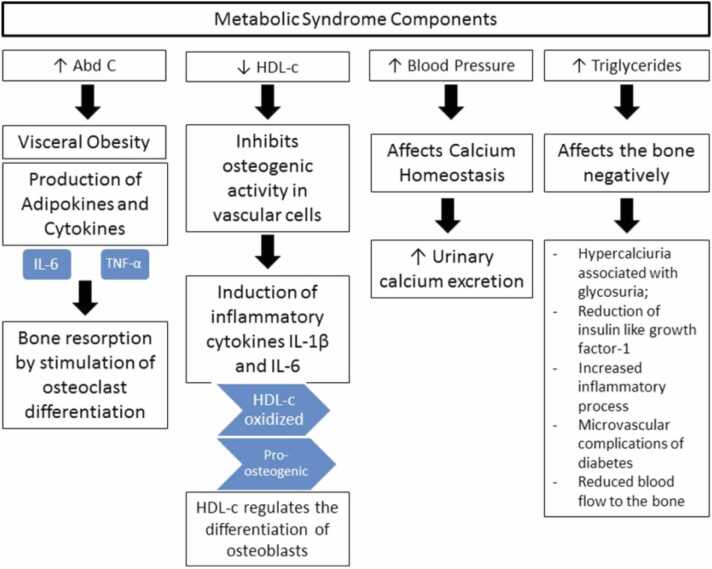
Chart showing the effects of the metabolic syndrome on skeletal physiology. Abd C, abdominal circumference; HDL-c, high-density lipoprotein c; IL, interleukin; TNF, tumor necrosis factor. Reproduced with permission from da Silva VN, Fiorelli LNM, da Silva CC, Kurokawa CS, Goldberg TBL: Do metabolic syndrome and its components have an impact on bone mineral density in adolescents? *Nutrition Metab*. 2017;14:1 [36].

Obesity is separated between total body/subcutaneous adiposity and central adiposity, also known as abdominal obesity. Abdominal obesity is determined by waist circumference and is defined as having a waist circumference >90th percentile for age and sex [33]. Central adiposity is the main culprit for the negative health effects seen in children and adults including the metabolic syndrome [27], [33] which leads to direct effects on the growing skeleton and generalized bone health (Fig. 1) [36].

Pediatric patients who are obese have increased mechanical load on their bones. They subsequently have larger bones than their normal-weight peers, greater bone strength, increased vertebral density, greater bone mineral content for height, increased height, and earlier maturation [27], [37], [38], [39]. Despite this, when obesity becomes a long-term condition, it imparts a systemic proinflammatory state that negatively affects bone health [40]. This proinflammatory state stems from endocrine-related changes created by obesity, and is further exacerbated by nutritional deficiencies leading to a lower ratio of overall bone mass when compared to a patient’s weight, lower total bone density, and lower bone mineral content [39], [41], [42]. This creates an environment that allows for low-velocity fractures as previously discussed. Peak bone mass attained in childhood affects bone health in adulthood [33] so modifying obesity-related factors in pediatric patients can impart long-term bone health benefits.

Bone health constitutes a flux in the bone turnover balance between formation and resorption. For adults, bone physiology is steadily coordinated and based upon the physiological needs for calcium at any given time. In children, the bones primarily reside in a bone formation state. Any factors that grossly affect this bone formation state will lead to a further bone health imbalance. Skeletal regulation is a complex chemical cascade with adipocytes, osteoclasts, and osteoblasts derived in lineage from mesenchymal stem cells (Fig. 1) [32], [33], [42]. Proinflammatory cytokines overproduced in chronic obesity alters the differentiation of mesenchymal stem cells from osteoblasts into adipocytes [32]. These proinflammatory cytokines include interleukin-6, tumor necrosis factor-α (TNF-α), and C-reactive protein (CRP) which go on to activate a variety of bone regulation pathways [32], [33], [42].

The theory that obesity leads to a chronic inflammatory state started when TNF-α was found to be elevated in obese mice [43]. Subsequently, increasing CRP levels were found to increase significantly as BMI increased, with odds ratio of an elevated CRP of 1.51 when BMI was 25 to <30 up to 9.3 times greater when BMI was ≥40 [44]. This further led to the discovery of leptin, an adipocyte-derived cytokine directly secreted from adipocytes. Adipose tissue is now recognized as a distinct endocrine tissue [45]. In a chronic obese state, adipocytes increase in size as a result of hyperinsulinemia, and once they reach a critical size, they apoptose and release proinflammatory cytokines and adipocytokines leading to a litany of effects on the human skeleton [32].

**Figure 2:**
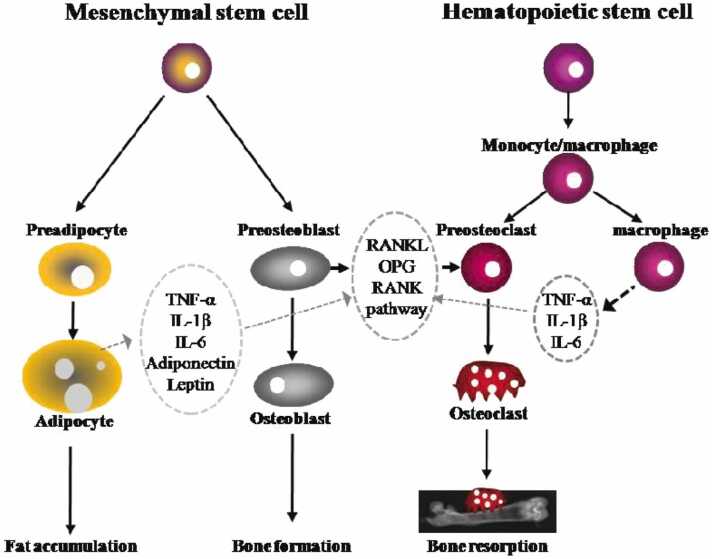
Bone metabolism regulated by adipocytes, osteoblasts, and osteoclasts. Fat accumulation is closely related to bone formation and resorption. Osteoblasts and adipocytes are derived from a common multipotential mesenchymal stem cell. Osteoclasts are differentiated from monocyte/macrophage precursors of hematopoietic stem cells origin. Adipocytes secrete several cytokines such as TNF-α, IL-1β, IL-6, adiponectin, and leptin which are capable of modulating osteoclastogenesis through RANKL/RANKL/OPG pathway. IL, interleukin; OPG, osteoprotegerin; RANK, receptor activator of nuclear transcription factor κB; RANKL, receptor activator of nuclear transcription factor κB ligand; TNF-α, tumor necrosis alpha. Reproduced with permission from Cao JJ. Effects of obesity on bone metabolism. *Journal of Orthopaedic Surgery and Research*. 2011;6:30 [42].

**Figure 3:**
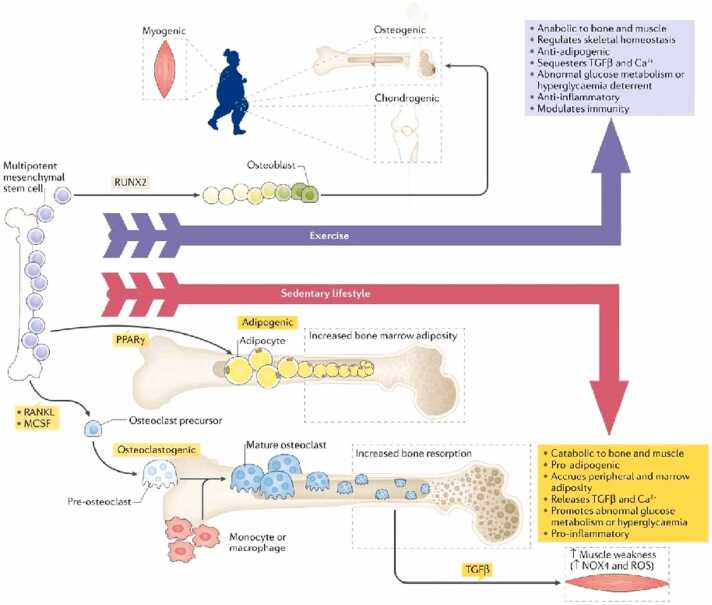
Impact of exercise and high-calorie diet and sedentary lifestyle on bone and fat. Mesenchymal stem cell lineage drives osteogenic differentiation. Exercise plays an important role in bone modeling and remodeling via the osteocyte network while suppressing the accumulation of fat. Sedentary and indoor lifestyles stimulate adipogenic programs accompanied by increased marrow adiposity. With poor mechanical load, osteocytes reduce osteoprotegerin levels inducing RANKL effects resulting in osteoclast-mediated resorption in bone. MCSF, mesenchymal stem cell factor; NOX4, NADPH oxidase 4; PPARγ, peroxisome proliferator-activated receptor gamma; RANKL, receptor activator of nuclear factor-κB ligand; ROS, reactive oxygen species; RUNX2, runt-related transcription factor 2; TGF-β, transforming growth factor-β. Reproduced with permission from Korkmaz HA, Ozkan B. Impact of obesity on bone metabolism in children. *J Pediatr Endocrinolo Metab*. 2022;35:557-565 [33].

The most well-known bone metabolism cycle is the nuclear factor kappa-B ligand (RANKL) and osteoprotegerin (OPG) pathway (Fig. 2). RANKL primarily and directly activates osteoclasts and bone resorption. OPG has a negative effect on RANKL activation. It binds to the RANKL-receptor, blocking RANKL from binding to its receptor, secondarily leading to inhibition of osteoclast formation. Increased body fat in obesity increases cortisol production which inhibits OPG production, leading to osteoclastogenesis [46]. Increased inflammatory cytokines (ie, include interleukin-6, TNF-α, CRP) released from apoptosed adipocytes lead to increased RANKL production and osteoclastogenesis [33]. Elevated TNF-α levels also inhibit osteoblastogenesis and cause osteoblast apoptosis [33]. For growing children, especially those with a high-fat diet and sedentary lifestyle, the native bone formation state will be negatively affected and lead to lower bone mineral density, regardless of size (Fig. 3).

**Figure 4:**
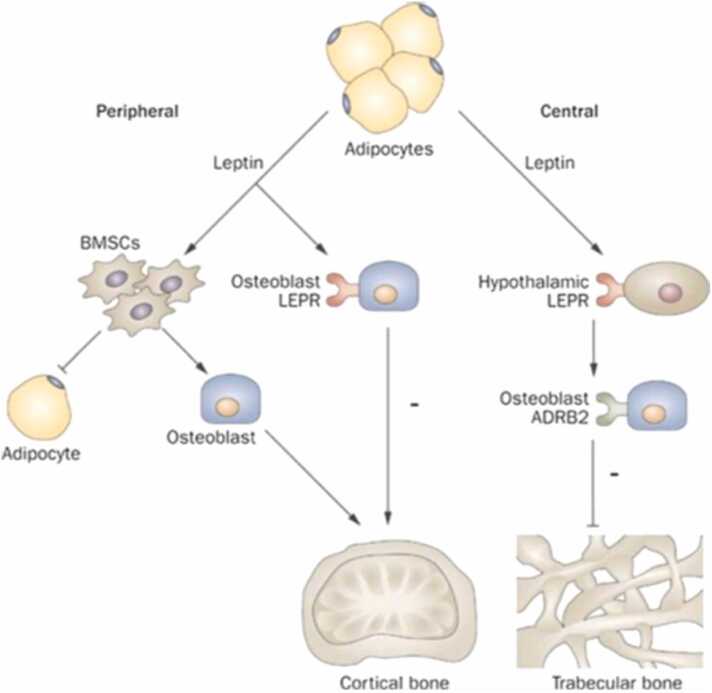
Illustration showing the mechanism of central and peripheral leptin signaling. ADRB2, B2 adrenergic receptor; BMSCs, bone marrow stromal cells; LEPR, leptin receptor. Reproduced with permission from Macmillan Publishers Ltd, Kawai M, Devlin MJ, Rosen CJ: Fat targets for skeletal health. *Nat Rev Rheumatol*. 2009;5:365-372 [53].

Leptin in its normal state is a beneficial hormone that regulates the human satiety center in the hypothalamus, assisting with appetite suppression and improving energy expenditure [47]. It also has bone health benefits by helping to activate osteoblast formation (Fig. 4) [48]. In obesity, leptin is abnormally increased with direct effects on bone production and resorption. Chronically elevated leptin levels cause leptin resistance which leads to inflammatory cytokine production and the bone effects discussed previously [49]. In addition, the leptin molecule in obese individuals is altered. Though it binds to its normal receptor, leptin in obese individuals does not carry the normal effect of binding to gonadotropin centers in the pituitary [33]. This in turn affects the gonadotropin (ie, estrogen and testosterone) effects on the skeleton, decreasing OPG production and increasing RANKL activation, leading to osteoclastogenesis and thus to decreased bone mass, decreased trabecular thickness, and increased cortical porosity [47], [50], [51], [52].

Adiponectin is leptin’s counterpart. Like leptin, it is secreted by adipocytes, but as opposed to leptin, it induces an anti-inflammatory effect on the body [54]. Adiponectin also helps to directly inhibit the production of osteoclasts and stimulate the production of osteoblasts [42], [50], [55], [56], [57], [58]. Adiponectin mainly targets liver tissue and skeletal muscle, acting to decrease glucose production, and increase insulin sensitivity as well as energy expenditure [59]. In obesity, adiponectin levels are decreased and lead to decreased osteoblast activity and bone formation [55], [60].

Vitamin D (25-OH vitamin D) is an important molecule for bone health and will be discussed in greater detail in other articles within this series. From the standpoint of obesity, vitamin D is fat-soluble and sequestered in adipose tissue. For obese patients, this decreases vitamin D availability in the circulation needed for proper bone health [39]. There is a high level of vitamin D deficiency in obese pediatric patients, reported to be present in 29% of overweight children, 34% of obese children, and 49% of severely obese children [61]. True vitaminD deficiency is not fully apparent in obese individuals. Free serum 25-OH vitamin-D levels could present as normal, but the total 25-OH vitamin D levels will be lower due to reduced levels of vitamin-D binding protein affected by adipokine production by adipocytes [33]. Hypovitaminosis D in obese patients is therefore likely underestimated. In general, poor diets are associated with obesity, such as found in “food deserts,” and add to the deficit of necessary micronutrients and nutrients such as vitamin D and calcium. Diets high in fat, carbonated sweetened drinks, and low in green leafy vegetables, lead to a higher risk for osteopenia [37]. High-fat diets can affect calcium absorption by creating calcium soaps in the intestine that increases calcium secretion rather than absorption [61], [62], [63], [64]. The stress hormone cortisol, which can lead to adipose tissue production, is increased when dietary calcium is low, further affecting fat metabolism and bone health [65].

Vitamin D helps to modulate the levels of leptin, adiponectin, and inflammatory cytokine production [66]. Through appropriate vitamin D and calcium supplementation, bone mineral density and total bone mass can be improved [67], [68]. When supplementing vitamin D, obese patients require doses 2 to 3 times higher than normal-weight patients, and overweight patients 1 and a half times higher than normal-weight individuals [25].

## Summary

Given vitamin D’s important role in pediatric bone health, identifying patients who are insufficient/deficient and properly improving their serum levels can optimize their peak bone health potentially preventing fractures both during childhood and throughout the rest of their lives. The overall effects of obesity on bone health are complex, but simple interventions such as appropriate nutrition and adequate exercise can help modulate obesity and its effects on the growing skeleton leading to improved long-term bone health as well as overall health.

## Additional links

• National Academies Press: Dietary Reference Intakes for Calcium and Vitamin D
• American Academy of Pediatrics: Clinical Practice Guideline for the Evaluation and Treatment of Children and Adolescents with Obesity: Clinical Practice Guideline for the Evaluation and Treatment of Children and Adolescents With Obesity

## Authors contributions

**Philip Nowicki:** Conceptualization, Writing – original draft, Writing – review & editing. **Adam Kreutzer:** Writing – original draft. **Jessica McMichael:** Conceptualization, Writing – original draft, Writing – review & editing.

## Declarations of competing interests

The authors declare the following financial interests/personal relationships which may be considered as potential competing interests: Philip Nowicki reports a relationship with OrthoPediatrics that includes consulting or advisory. Philip Nowicki-Editorial Board Member (Orthopaedics, Slack Inc). If there are other authors, they declare that they have no known competing financial interests or personal relationships that could have appeared to influence the work reported in this paper.
